# Abstract of an Address Delivered before the Alumni Association of Rush Medical College

**Published:** 1880-12

**Authors:** DeLaskie Miller

**Affiliations:** Acting President of the College


### ARTICLE V.

ABSTRACT FROM AN ADDRESS DELIVERED BEFORE THE ALUMNI ASSOCIATION OF RUSH MEDICAL COLLEGE, at their annual banquet, in the Grand Pacific Hotel, Chicago, Feb. 25, 1880. By PROF. DELASKIE MILLER, Acting President of the College.

"Know thou thyself" is an admonition which has passed currently among men for many generations, and it may be accepted as genuine coin to-day, for self-knowledge is undoubtedly invaluable. But how may a man know himself? This query involves a problem not easily solved by many.

A short time since I was accosted by a gentleman who introduced himself by a name with which I had long been familiar, even from early boyhood. I had not seen him for many years. I said to him: "I should know you. I recall many incidents connected with your personal history, but the change in your external appearance is so great that I could not identify you. When you think of it, the change must seem as great to you as it appears to me. You are now justly regarded as a statesman. You are the chief executive of your State. How do *you* know that you are the same person with whom I attended the primary school and played at games in early life? There is not one particle of matter in your system now that composed your body then." His answer was philosophical and satisfactory. He replied, "By *memory* and my *consciousness* I have proof of my identity."

It is not difficult to perceive the parallel in the history of the individual and the development and growth of a great institution. Who that has seen Rush Medical College to-day could realize that *it is* the same institution that originated on Clark street? Then, a few plain seats in a rear room of an inferior building accommodated the students in attendance upon the lectures—now, nearly five hundred seats are occupied in a building which would be an ornament to any city. Then, two Professors gave the entire course of lectures—now, more than thirty enthusiastic physicians are engaged in teaching the classes. Then, sixteen weeks of each year was considered ample time in which to qualify young men for the responsibilities of medical practice—now, systematic instruction is given during thirty-seven weeks of each college year. Then, *clinics* constituted no part of the curriculum—now, students are *required* to attend the clinics which are given daily throughout the term. Then, practical chemistry was not thought of as an exercise for the student—now, practical work in the laboratory is considered important, and many students become proficient there.

In another respect I can trace the analogy in the history of the individual and the college. As in man the elements are constantly changing, so is it with the institution. Herrick was laid to rest in an Eastern city. Brainard ceased from his labors in the midst of a prosperous course. Blaney was called home after he had attained the highest honors of the institution; and Freer, of blessed memory, has ascended to his reward. Thus there is not one left who assisted in the organization and early history of the college. But no—I must qualify that statement—not one of the early faculty remains. There is one honored name still with us—the name of a gentleman who took an active part in the organization of the college, and who has ever felt and manifested a lively interest in its prosperity—who has faithfully discharged the duties of secretary of the board of trustees from the first meeting even until now, and whose name is attached to the diploma of each alumnus of the college from its organization. As Judge Goodrich is unable to be present this evening, I give the alumni present his cordial greeting.

Gentlemen, we hear much said during these latter years upon the subject of medical education. And much that we hear is anything but commendatory of the system now in vogue. There are in our ranks some who may be styled pessimists—who believe the present time just about as bad as it well could be—who think the medical teaching of to-day anything but thorough—who feel that the profession has deteriorated by the admission of young men, since they graduated. Now, I do not sympathize with these sentiments in any degree. I believe in optimism. I believe the present time better than any former time. The medical profession holds a more honored position to-day than ever before. It exercises a greater influence for good. It is consulted with more confidence on important questions involving the highest interests of society than in any past time.

Am I warranted in assuming that Rush Medical College has contributed a share to this advancement—this elevation of the profession? That she has done some faithful work during these thirty-six years of her active existence? Who fill many of the important chairs in the medical colleges in this city and in different sections of this country? Graduates of Rush Medical College. Who is at the head of the Marine Hospital Service of the United States? A graduate of Rush Medical College. But I would weary you were I to call the roll of honor. I claim that the past history of the college must be accepted as prophetic of her future increasing influence and usefulness.

It will not be expected that I should enumerate the improvements which have been inaugurated by Rush Medical College. I may, however, refer to the mode adopted for filling vacancies in the Faculty by *concours*—a mode which has proved highly satisfactory; a mode which has attracted some of the best talent in the land; a mode which holds out inducements in the future to the graduates of this, the *best* class, and of which they will undoubtedly eagerly avail themselves.

During all the changes—internal and external—which have marked the career of the college, her progress has been onward and upward. Though her Faculty, some of them giants in intellect, may by the inscrutable providence of God, be removed from the sphere of their usefulness here—though the material fabric may be dissipated in thin air and disappear in smoke—still the college survives, for she is animated by a living soul—she is stimulated by the same spirit now as heretofore. She is not averse to improvement—she is not traveling in a rut. She is constantly augmenting the means and facilities for greater thoroughness in teaching—she is constantly exacting higher attainments of her graduates. She is determined to keep abreast with the most advanced in every real improvement.

As long as the right material for our classes is furnished by the profession of the country, so long will "old Rush" continue to add to the profession physicians who will take rank with the representative men who are present to-night.

---

A REMEDY FOR THE SICK-STOMACH OF PREGNANCY.—Doctor Forwood, at a late meeting of the Lancaster, Pa., Medical Society, read an interesting paper on the "Treatment of the Sick-Stomach of Pregnancy." His favorite prescription is as follows:

℞ Rad. columbo..............................
Rad. zingiber, āā.......................... ℥ss.
Fol. sennæ.................................. ʒj.
Aquæ bullient ............................ Oj.
Mix. Infus..................................
Sig. Take a wineglassful before each meal.

Dr. Forwood says: "For a time we used these medicines in a powdered state, but finally abandoned it in that form because of the 'mushy' character of the infusion, which was disagreeable to the patient and difficult to filter with ordinary appliances."

